# Occurrence of Trifluoroacetic Acid in Wine and Its Relevance for Dietary Exposure and Human Health: A Narrative Review

**DOI:** 10.3390/toxics14060454

**Published:** 2026-05-22

**Authors:** Andrea Moscato, Paola Rapisarda, Margherita Ferrante, Maria Fiore

**Affiliations:** 1 School of Specialization in Hygiene, Department of Medical, Surgical Sciences and Advanced Technologies “GF Ingrassia”, University of Catania, Via S. Sofia 87, 95123 Catania, Italy; moscatoandrea99@gmail.com; 2 Department of Medical, Surgical Sciences and Advanced Technologies “GF Ingrassia”, University of Catania, Via S. Sofia 87, 95123 Catania, Italy; paola.rapisarda@phd.unict.it (P.R.); marfer@unict.it (M.F.)

**Keywords:** trifluoroacetic acid, PFAS, environmental persistence, human exposure, wine contamination, agricultural pathways, toxicological evidence

## Abstract

Trifluoroacetic acid (TFA) is an ultrashort-chain perfluoroalkyl substance (PFAS) characterized by environmental persistence, water solubility, and a growing global presence, resulting primarily from the degradation of fluorinated compounds. Evidence suggests that plant-based foods may represent an underestimated exposure route, with wine emerging as a significant dietary source due to accumulation in soils, irrigation water, and plant uptake. This review provides an updated summary of the evidence on the environmental sources and temporal evolution of TFA in wine, its analytical detection, its contribution to dietary exposure, potential implications for human health, and current regulatory attention. A structured but non-systematic literature search was conducted using PubMed and Scopus, supplemented by European reports and monitoring data, and in accordance with SANRA guidelines. Evidence shows that TFA concentrations in wine derive from widespread environmental sources and have increased over time, from negligible levels before the 1970s to a marked increase in recent decades. Reported concentrations range from tens to several hundred µg/L, despite analytical challenges. Exposure estimates indicate that wine may contribute significantly to total dietary TFA intake in regular consumers. Although toxicological data suggest low acute toxicity, uncertainties remain regarding long-term exposure, and regulatory limits for TFA in foods and beverages are lacking.

## 1. Introduction

Trifluoroacetic acid (TFA) has recently emerged as one of the most pervasive and persistent environmental contaminants worldwide [1]. Although classified among the ultrashort-chain per- and polyfluoroalkyl substances (PFAS), which are traditionally considered less hazardous than long-chain analogues, TFA exhibits a unique combination of extreme persistence, high mobility, and a remarkable capacity to accumulate in environmental and biological systems [2]. Growing evidence indicates a continuous rise in TFA concentrations across multiple environmental compartments, including surface and groundwater, precipitation, soils, plants, food products, and even human serum, revealing a pattern of widespread and steadily increasing exposure [3,4,5].

This global accumulation is driven by a wide range of sources. TFA is a terminal degradation product of numerous fluorinated refrigerants (F-gases), several widely used fluorinated pesticides, pharmaceuticals, industrial chemicals, and fluoropolymers [6,7]. Additional contributions arise from industrial emissions, wastewater treatment, landfills, and thermal destruction processes involving PFAS-containing materials [8,9]. Due to the absence of effective natural degradation pathways and the limited efficiency of available removal technologies, TFA behaves as an essentially irreversible contaminant, progressively accumulating in water resources and biota over time [10,11].

Parallel to its environmental expansion, emerging toxicological evidence has raised questions regarding possible developmental, reproductive, and hepatic effects, although current risk assessments generally do not indicate an immediate health concern at present environmental exposure levels [12]. Ecotoxicological data, though still incomplete, indicate notable sensitivity among aquatic organisms and plants, raising concerns about long-term ecosystem impacts. Given its persistence, mobility, and multigenerational exposure potential, TFA is increasingly recognized as a contaminant of environmental and regulatory concern [13].

Moreover, in recent years, wine has increasingly been recognized as a potential dietary source of TFA, reflecting the compound’s growing accumulation in agricultural soils, irrigation water, and atmospheric deposition [14]. Because grapevines readily take up ultrashort-chain PFAS from soil and water, TFA can be transferred into grapes and subsequently into wine, contributing to chronic low-dose human exposure. Although the toxicological profile of TFA is still being refined, the combination of extreme environmental persistence, increasing occurrence, and remaining uncertainties regarding chronic low-dose exposure supports the need for continued monitoring and further toxicological evaluation, including for commonly consumed beverages such as wine [15]. Assessing TFA levels in wine is therefore essential to better understand its contribution to overall exposure and to evaluate potential public health implications within the broader context of persistent and mobile PFAS [16].

Internationally, TFA is increasingly recognized as a persistent and mobile PFAS of emerging regulatory concern. Within the European Union, TFA is evaluated under the Persistent, Mobile, Toxic (PMT) or very Persistent, very Mobile (vPvM) framework due to its extreme persistence, high mobility, and widespread occurrence in water resources [17]. Germany has proposed classifying TFA as a Category 1B reproductive toxicant under the CLP Regulation, a designation that, if adopted, would have significant implications for its management across environmental and food-related matrices. Several Member States have already established drinking water guideline values: Germany has set a reference value of 60 µg/L (with a precautionary target of 10 µg/L), while the Netherlands has proposed an indicative limit of 2.2 µg/L. Moreover, the recast EU Drinking Water Directive includes a 0.5 µg/L limit for the sum of all PFAS, a threshold that TFA alone exceeds in many European water sources [18,19,20].

At present, no specific regulatory limits exist for TFA in food or beverages, including wine, highlighting an area where further exposure assessment and monitoring are needed. Nevertheless, the compound’s environmental ubiquity, rising concentrations, and remaining toxicological uncertainties are prompting growing attention from regulatory bodies. Overall, the evolving regulatory landscape underscores the need for systematic monitoring of TFA in agricultural products and a more comprehensive evaluation of its contribution to total human exposure [2].

This narrative review aims to critically synthesize current evidence on trifluoroacetic acid (TFA) in wine, with particular focus on its environmental sources and temporal evolution, occurrence, spatial distribution, analytical detection across wine-producing regions, comparison with other beverages and plant-derived food matrices, relevance for dietary exposure, potential implications for human health, and current regulatory attention.

## 2. Materials and Methods

This narrative review was conducted in accordance with the SANRA (Scale for the Assessment of Narrative Review Articles) guidelines, which are specifically designed to support the methodological quality, transparency, and critical appraisal of narrative reviews.

### 2.1. Literature Search Strategy and Source Selection

The literature search was conducted using two major biomedical and scientific databases, PubMed/MEDLINE and Scopus. The main search focused on literature published between January 2016 and December 2025, in order to capture the most recent developments concerning TFA occurrence, analytical methods, environmental fate, toxicological evidence, dietary exposure, and regulatory attention. Earlier studies were also included when they provided foundational information on environmental occurrence, analytical detection, toxicological assessment, or historical trends relevant to the interpretation of TFA contamination in wine.

The search strategy combined free-text keywords and Boolean operators (“AND”, “OR”) to maximize sensitivity and specificity. The following search terms and combinations were used and adapted according to the syntax of each database: (“trifluoroacetic acid” OR “trifluoroacetate” OR TFA OR PFAS OR “perfluoroalkyl substances”) AND (“environmental persistence” OR persistence OR degradation OR “environmental fate”); (“trifluoroacetic acid” OR TFA) AND (“human exposure” OR “dietary exposure” OR biomonitoring); (“trifluoroacetic acid” OR TFA) AND (wine OR “wine contamination” OR beverages OR food); (“trifluoroacetic acid” OR TFA) AND (“agricultural pathways” OR agriculture OR crops OR irrigation OR soil OR “plant uptake”); and (“trifluoroacetic acid” OR TFA) AND (toxicology OR toxicity OR “toxicological evidence” OR “risk assessment” OR regulation OR REACH OR ECHA).

Reference lists of relevant articles and reports were also screened to identify additional sources. Selected technical reports, regulatory documents, and monitoring datasets were included when they provided essential information not yet available in the peer-reviewed literature, particularly regarding recent occurrence data, regulatory thresholds, and monitoring evidence for TFA in wine and related environmental matrices.

### 2.2. Eligibility Criteria and Source Appraisal

We included original research articles, reviews, technical reports from regulatory agencies, REACH documents, and national and international guidelines, published in English and relevant to the topics addressed in this review. Selection was based on relevance, prioritizing recent studies and those with reliable quantitative data. Studies were included if they met at least one of the following criteria:

- Reported measured concentrations of TFA in wine, grapes, or related plant-based beverages;
- Investigated environmental levels of TFA in matrices relevant to viticulture (soil, irrigation water, precipitation, atmospheric deposition);
- Examined plant uptake mechanisms or transfer of ultrashort-chain PFAS into edible crops;
- Provided toxicological, toxicokinetic, or biomonitoring data relevant to human exposure to TFA;
- Addressed regulatory aspects, risk assessment, or environmental policy related to TFA or ultrashort-chain PFAS.

Articles were excluded if they did not provide primary data or lacked scientific relevance, such as opinion pieces without empirical evidence. We also excluded analytical studies in which TFA was used solely as a laboratory reagent without any environmental or exposure-related implications. In addition, duplicates, conference abstracts without full text, and non-peer-reviewed sources were excluded unless they contained essential regulatory, monitoring, or technical information. Because peer-reviewed occurrence data on TFA in wine remain scarce, selected grey literature sources, including regulatory documents, technical reports, and monitoring datasets, were considered when they provided relevant and timely information not yet available in the peer-reviewed literature. These sources were not treated as equivalent to peer-reviewed studies but were used as contextual and supporting evidence. Particular attention was paid to the transparency of the source, the description of sampling procedures, analytical methods, quality-control information, and consistency with peer-reviewed evidence. When methodological details were incomplete, this uncertainty was explicitly acknowledged and the findings were interpreted cautiously. Sources were managed using Zotero for citation organization and bibliography generation. Given the heterogeneity of analytical methods, matrices, reporting formats, detection limits, recovery rates, and matrix-effect correction procedures, no quantitative meta-analysis or statistical standardization of concentration data was performed.

As this was a narrative review, no pre-registered protocol was followed, nor was a systematic risk of bias assessment performed. However, particular attention was given to the methodological quality of the included studies, the consistency of the results, and the triangulation of the sources, with specific reference to the SANRA domains [21], including:

- Justification of the review’s relevance;
- Clarity of aims;
- Adequacy of literature search;
- Scientific reasoning and balance;
- Appropriate referencing;
- Transparent presentation of limitations.

This review did not involve human subjects, animals, or confidential data and therefore did not require ethical approval.

## 3. Results and Discussion

### 3.1. Environmental Sources and Historical Evidence on the Temporal Evolution of TFA Levels in Wine

Trifluoroacetic acid (TFA) is now regarded as one of the most ubiquitous and environmentally persistent transformation products within the broader PFAS family [1]. Its environmental presence does not originate from a single source but reflects the cumulative degradation of numerous anthropogenic fluorinated compounds used across industrial and commercial sectors. These include hydrofluorocarbon and hydrofluoroolefin refrigerants, fluorinated agrochemicals, selected pharmaceutical compounds, specialty industrial products, and fluoropolymer-based materials. Additional environmental inputs arise from diffuse emissions linked to manufacturing processes, wastewater treatment effluents, landfill leachates, and the thermal treatment of PFAS-containing waste streams [22].

A defining feature of TFA is its exceptional chemical stability combined with high mobility in aqueous environments. The compound is highly resistant to both biotic and abiotic degradation, and existing water treatment technologies are only partially effective in removing it. Consequently, TFA behaves as a long-lived and effectively irreversible contaminant that is continuously redistributed through the hydrological cycle, progressively enriching surface and groundwater systems and facilitating its transfer into terrestrial and aquatic food webs [23].

When wine-specific evidence is integrated with this broader environmental framework, a plausible model of contamination along the vitivinicultural chain emerges. Due to its high water solubility and persistence, TFA deposited via precipitation or introduced into soils through contaminated irrigation water can be efficiently absorbed by plants [24,25]. Experimental evidence indicates that TFA exhibits a comparatively high root-uptake factor relative to other short-chain PFAS, with a marked tendency to accumulate in plant tissues. Grapevines, as perennial crops with multi-decade productive cycles, are therefore particularly susceptible to the progressive buildup of TFA over time.

The detection of TFA even in organic wines, albeit sometimes at lower concentrations than in conventional products, supports the interpretation that atmospheric deposition constitutes an unavoidable background source of contamination [26]. In addition, European monitoring data suggest a potential association between elevated TFA concentrations and the concurrent presence of synthetic pesticide residues, indicating that certain agricultural practices may indirectly contribute to contaminant accumulation through the use of substances containing fluorinated precursors. Soil is a crucial environmental compartment for TFA pollution.

Compared with other environmental compartments, soils generally have higher TFA levels than air or water. Reported atmospheric concentrations typically fall in the low ng/m^3^ range, while precipitation contains TFA at levels below ng/mL. Surface waters exhibit slightly wider variability, generally below 1 ng/mL, and groundwater concentrations are typically even lower, in the range of a few pg/mL [27,28,29,30]. In contrast, soils often contain TFA in the ng/g range, commonly between about 0.2 and 14 ng/g. Experimental observations indicate that TFA behavior in soils is more complex than expected. Even in samples with relatively low levels of contamination, TFA showed significant retention within the solid matrix. This finding suggests that specific interactions with soil components, such as mineral surfaces or organic matter, may partially limit their leaching potential. Such retention mechanisms may contribute to the relatively low concentrations detected in groundwater [31].

Available data on terrestrial plants, reporting concentrations between 1 and 4 ng/g, further support the role of soil as a key intermediary in the transfer of TFA within terrestrial ecosystems [32]. The fact that levels in plants exceed those typically observed in surface waters strengthens the hypothesis that soil-associated TFA represent an important source of exposure for plants, highlighting soil as a critical compartment in the environmental distribution and potential transfer of this compound into the food chain. Together, these processes position wine as an environmental integrator that reflects both diffuse atmospheric inputs and agricultural influences within the broader cycle of TFA contamination.

**Figure 1:**
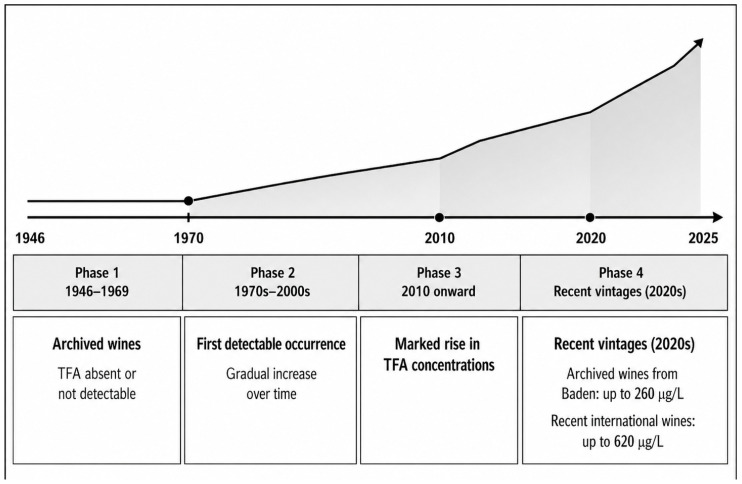
Historical evolution of TFA occurrence in wine based on archived-wine evidence.

A key contribution to the understanding of historical trends is provided by the retrospective archived-wine study Tracking Trifluoroacetate (TFA) through Time, which analyzed a unique collection of archival wines produced between 1946 and 2024 [33]. This work makes it possible to reconstruct the temporal evolution of TFA contamination in wine over a period of nearly eighty years, using highly specific analytical methods and rigorous contamination controls. The results show that TFA is not detectable in wines produced before the 1970s, whereas from that period onward it appears in progressively increasing concentrations. The rise becomes particularly pronounced after 2010, with archived wines from Baden reaching up to 260 µg/L and recent international wines reaching up to 620 µg/L, as summarized in Figure 1 [33].

This temporal pattern is consistent with the global increase in the use of fluorinated compounds, especially refrigerant gases (HFCs and HFOs) and pesticides containing trifluoromethyl groups, which are known precursors of TFA. The role of fluorinated pesticides as potential TFA precursors is increasingly supported by food surveillance data. In a long-term analysis of 6034 food samples marketed in Luxembourg between 2011 and 2024, fluorinated pesticide residues were detected in 12.3% of samples, with detection rates increasing from 9.6% in 2011 to 26.8% in 2024. Thirty-one distinct fluorinated pesticides were identified, and six compounds (fluopyram, lambda-cyhalothrin, trifloxystrobin, bifenthrin, fluopicolide, and flonicamid) accounted for nearly 80% of detections. These active substances are considered potential precursors of TFA formation [34]. Although these data are not specific to wine or viticulture, they support the broader concern that fluorinated pesticide use in agriculture may contribute to environmental TFA formation and subsequent transfer into food matrices.

The study also suggests that TFA occurrence in wine is not restricted to a single geographical context. However, because available wine-specific data remain unevenly distributed across regions, current evidence should not be interpreted as providing fully representative global estimates. Rather, these findings indicate that TFA contamination in wine may reflect broader environmental accumulation processes, while highlighting the need for more harmonized monitoring across both European and non-European wine-producing regions. Wine may therefore represent a useful environmental archive, capable of reflecting the progressive accumulation of TFA in terrestrial environmental compartments [33].

### 3.2. Reported Concentrations, Spatial Distribution and Analytical Detection of TFA Across Wine-Producing Regions

Recent scientific evidence suggests that wine may be among the plant-derived beverages with relatively high reported concentrations of TFA. Although the number of peer-reviewed studies specifically dedicated to TFA in wine is still limited, the available data are consistent in suggesting measurable TFA occurrence in wine, although geographical coverage remains limited and uneven. A key contribution comes from the study by Affricano et al., which applied a targeted HPLC–MS/MS methodology for the analysis of ultrashort-chain PFAS in water and beverages, including wine samples. The results show that TFA is the only compound of this class systematically detected, and that its concentrations in wines are markedly higher than those measured in drinking water analyzed [24]. In the same study, Italian wine samples showed substantially higher TFA levels than water samples. Mean concentrations were 130.89 µg/L in wine compared with 0.39 µg/L in Italian water samples, while red and white wines showed mean values of 144.30 µg/L and 116.32 µg/L, respectively. Several red wine samples exceeded 300 µg/L, further supporting the interpretation that wine may represent a relevant dietary matrix for TFA exposure in wine-consuming populations [24].

Because reported concentrations derive from studies using different analytical platforms, sample preparation procedures, limits of detection and quantification, recovery rates, and approaches to matrix-effect correction, the values summarized in this review should be interpreted as indicative concentration ranges rather than as directly comparable pooled estimates. Specifically, while water samples generally exhibit concentrations below a few µg/L, wines have been reported to show levels in the range of tens to hundreds of µg/L. This observation suggests that wine may not simply reflect water contamination alone, but may act as an integrative matrix of environmental exposure to TFA throughout the grapevine cultivation cycle.

Additional evidence supporting the widespread environmental occurrence of TFA comes from studies on ultra-short-chain perfluoroalkyl acids (PFAAs) in water systems. In one investigation, TFA was consistently detected in samples collected near potential point sources such as landfills, firefighting training sites, and hazardous waste facilities, reaching concentrations up to 14,000 ng/L. These findings highlight the ubiquity of TFA in aquatic environments and reinforce its role as a persistent and mobile contaminant capable of entering agricultural systems and, consequently, the food chain [35]. The Italian study also included a small set of Asian water samples for preliminary comparison. Thai bottled and tap waters showed no detectable or quantifiable TFA levels, whereas Chinese bottled, tap, and surface water samples showed measurable concentrations of 0.56, 0.85, and 1.85 µg/L, respectively. However, these data refer to water samples only and should not be interpreted as evidence of TFA occurrence in Asian wines [24].

Additional contextual evidence is provided by the European monitoring report “Message from the Bottle—The Rapid Rise of TFA Contamination Across the EU”, which examined 49 European wines, including historical bottles and recent vintages. Although this report was not published as a peer-reviewed study and provides limited publicly available information on analytical quality-control procedures, its findings are consistent with the temporal pattern reported in peer-reviewed archival wine analyses, showing the absence or very low detection of TFA in older wines and measurable concentrations in recent vintages. Therefore, these data were considered as supportive monitoring evidence rather than as definitive quantitative estimates [36].

The sampling covered several European wine-producing countries, including France, whereas Italy was not represented. Although some differences between countries were reported, the limited sample size and non-representative sampling design do not allow robust country-level comparisons. Therefore, these findings should be interpreted as preliminary monitoring evidence rather than as representative national estimates. They further highlight the need for broader, harmonized, and peer-reviewed monitoring across both European and non-European wine-producing regions [37].

Recent Italian data have also raised the possibility that production-chain factors may influence TFA levels in finished wine. Lower concentrations were observed in supermarket low-cost wines than in winery-bottled wines, leading to the hypothesis that standardized industrial processes, such as filtration or stabilization, and differences in irrigation sources may affect residual TFA levels. However, these observations remain exploratory, and direct evidence quantifying the contribution of winemaking processes to TFA contamination is currently lacking [24].

The determination of trifluoroacetic acid (TFA) poses significant analytical challenges due to its small molecular size, high polarity (log P_OW_ ≈ −2.1) and very low pK_a_ (0.2–0.5), which result in complete dissociation in aqueous media. These properties lead to poor retention in conventional reversed-phase LC methods commonly applied for long-chain PFAS, where TFA often elutes in the dead volume, compromising sensitivity and selectivity [31].

As highlighted in recent method-development studies, analytical constraints have historically limited reliable quantification of TFA in environmental matrices [38]. Early analytical approaches for TFA in soils and environmental samples relied on gas chromatography–mass spectrometry (GC–MS) following derivatization, including esterification or reactions with fluorinated diazo compounds. While effective, these procedures are labor-intensive and prone to contamination and blank issues [31].

More recently, liquid chromatography coupled to tandem mass spectrometry (LC–MS/MS) has become the preferred approach; however, methods adapted from long-chain PFAS protocols are often inadequate for TFA due to insufficient chromatographic retention.

To overcome these limitations, dedicated separation strategies have been introduced. The use of hydrophilic interaction liquid chromatography (HILIC) or ion chromatography (IC) coupled with MS/MS detection has proven particularly effective. An optimized HILIC–MS/MS method for soil analysis achieved limits of detection as low as 0.015 ng/g and limits of quantification of 0.045 ng/g using isotope dilution with ^13^C_2_-TFA as internal standard [31].

Similarly, a rapid non-suppressed ion chromatography–MS/MS (nsIC–MS/MS) method enabled direct injection analysis of Antarctic ice cores with a limit of detection of 0.3 ng/L and high repeatability (<7%), demonstrating the suitability of anion-exchange–based separations for ultra-trace analysis [39].

Sample preparation remains a critical step, particularly for complex matrices such as soils and plant-derived products. Comparative extraction studies have shown that water- or carbonate-based extraction combined with isotope dilution provides more reliable recoveries than simple methanol-based protocols adapted from PFAS multi-residue methods [31].

Moreover, the strong mobility of TFA in soil–plant systems and its ubiquitous environmental presence underscore the need for sensitive and harmonized analytical methodologies [38].

Overall, accurate monitoring of TFA requires matrix-specific extraction procedures, dedicated anion-exchange or HILIC separations, isotope-labeled internal standards, and rigorous blank control. These methodological refinements are essential to generate robust occurrence data in environmental and food matrices, including wine, and to support reliable dietary exposure assessment. Until harmonized analytical protocols become available, comparisons across studies should be interpreted cautiously, and concentration data should not be considered directly interchangeable.

### 3.3. Estimated Dietary Exposure to TFA Attributable to Wine Consumption

From a public health perspective, the presence of TFA in wine becomes particularly relevant when considered in the context of the reference thresholds proposed for drinking water. In Germany, the health-based guidance value for TFA in water is 60 µg/L, with a precautionary target of 10 µg/L; in the Netherlands, an even more stringent value of 2.2 µg/L has been proposed. Reported concentrations in wine may exceed these drinking-water reference values, although such values should be interpreted only as contextual benchmarks and not as regulatory limits for wine [32,40].

This suggests a possible combined effect of the vine’s prolonged environmental exposure and the concentration processes occurring along the winemaking supply chain. Wine may therefore represent a relevant dietary contributor to TFA intake among commonly consumed beverages, particularly in regular consumers and in countries with high per capita wine consumption [41].

Assuming an average TFA concentration in wine of approximately 120 µg/L, a moderate consumption of 150 mL/day would result in a daily intake of about 18 µg of TFA, whereas a higher consumption of 300 mL/day would lead to an intake of roughly 36 µg/day. In some exposure scenarios, using the highest reported values (>300 µg/L), daily intake could exceed 45–90 µg [42].

Although these values remain below the oral DNELs proposed under the REACH regulation, it is important to emphasize that wine represents only one of multiple sources of TFA exposure. Cumulative intake from drinking water, other beverages, and plant-derived foods may lead to continuous chronic exposure that is not adequately captured by risk assessments focusing on individual matrices [43].

### 3.4. Wine Compared with Other Beverages and Plant-Derived Food Matrices

The comparison between wine and other beverages suggests that wine may represent a relevant dietary contributor to TFA intake, although it should not be considered a unique source of exposure. Recent evidence indicates that TFA has been detected in several plant-derived beverages, including beer, tea, herbal infusions, fruit juices, nectars, and wine, with higher reported concentrations in recent wine samples than in several other beverage matrices (Table 1). In beer samples from 23 countries, concentrations up to 51 µg/L were reported, with a median value of 6.1 µg/L; tea and herbal infusions showed a median concentration of 2.4 µg/L, suggesting unintended aqueous extraction from plant-derived raw materials [23].

In comparison, recent wines have shown markedly higher reported concentrations, with archived wines from Baden reaching up to 260 µg/L and recent international wines reaching up to 620 µg/L. Fruit juices and nectars were also included in comparative analyses, supporting the interpretation that wine may show higher TFA levels than several other plant-derived beverages [33]. However, comparisons across matrices should be interpreted cautiously because of differences in sampling design, analytical methods, geographical origin, detection limits, and production processes.

Overall, current evidence suggests that wine may be among the plant-based beverages with higher reported TFA concentrations, particularly in recent vintages. Nevertheless, available data remain limited, and further comparative multi-matrix studies including wine, beer, tea, herbal infusions, fruit juices, bottled water, and tap water are needed to clarify the relative contribution of different beverages to total dietary TFA exposure.

### 3.5. Potential Implications for Human Health

From a toxicological standpoint, TFA is generally characterized by low acute toxicity and the absence of lipid bioaccumulation. After oral intake, it is rapidly absorbed, distributed primarily within extracellular fluids, and excreted largely unchanged via the kidneys. Although its biological half-life is relatively short, continuous low-level exposure may occur in the context of daily dietary intake and environmental contamination [12].

Experimental repeated-dose studies in animals identify the liver as the principal target organ, with mild hepatocellular hypertrophy reported at higher chronic doses. Biomarker data suggest that TFA acts as a weak peroxisome proliferator in rodents, but it has not demonstrated genotoxicity or adverse effects in developmental or extended one-generation reproductive studies. Overall, margins of exposure (MoE) calculated from current concentrations in water and diet are considered sufficiently large (>100), indicating no immediate health concern under present exposure scenarios [44].

Most human clinical data derive from the historical use of halothane anesthesia. Halothane is metabolized via cytochrome P450 oxidation to TFA, bromide, and reactive intermediates capable of forming protein adducts in the liver. Severe “halothane hepatitis” has been attributed to immune-mediated reactions against these modified proteins rather than to TFA itself. Pharmacokinetic studies in this context demonstrated transient accumulation of TFA in serum after repeated halothane exposure, with partial enterohepatic recirculation contributing to delayed elimination. Importantly, these exposure conditions, primarily inhalational and at doses substantially higher than those expected from environmental sources, differ markedly from chronic low-dose dietary intake [44].

Case reports involving accidental contact with concentrated TFA describe corrosive injuries consistent with its strong acid properties. However, such effects relate to local chemical burns and are not representative of systemic toxicity at environmentally relevant concentrations [45]. Only very limited data are available regarding repeated occupational exposure; one report described dermatitis after prolonged laboratory exposure to airborne TFA, but no consistent systemic clinical outcomes have been documented [46].

Human biomonitoring studies indicate widespread, low-level exposure. TFA has been detected in serum, urine, and cord blood samples, although available datasets are insufficient to derive reliable estimates of dietary intake or long-term risk. Recently reported large-scale pooled urine analyses confirmed ubiquitous detection, with concentrations spanning a broad range, reflecting variable exposure patterns. Nevertheless, epidemiological investigations specifically addressing chronic dietary exposure, particularly through foods and beverages such as wine, are currently lacking [47,48].

In summary, current toxicological evidence indicates that TFA has low acute toxicity, no clear genotoxic potential, and limited systemic hazard at present exposure levels. Although wine-related TFA exposure does not currently represent a clearly established health risk, further research on chronic low-dose and cumulative exposure is needed to refine risk assessment.

### 3.6. Regulatory Attention to Persistent Fluorinated Contaminants

As shown in Table 2, the European Union has not yet established a specific legally binding limit for trifluoroacetic acid (TFA). However, several national authorities and regulatory frameworks have proposed guidance and benchmark values to contextualize exposure risks.

In Germany, a health-based reference value was introduced in 2020, setting a guidance concentration of 60 µg/L for TFA in drinking water, derived from chronic toxicity data obtained in animal studies. In addition, German authorities recommended a precautionary approach, suggesting that concentrations should ideally remain below a target value of 10 µg/L whenever technically feasible [49].

A more conservative provisional benchmark was later proposed in the Netherlands, where a tentative value of 2.2 µg/L was calculated by applying a relative potency factor referenced to perfluorooctanoic acid (PFOA) and its established drinking water safety threshold [50].

Within the framework of the REACH regulation, risk assessment has so far focused primarily on oral exposure. The derived no-effect level (DNEL) for the general population has been set at 0.042 mg/kg body weight per day, and current evaluations have not identified significant concerns associated with alternative exposure routes [43,51,52]. At the European policy level, the recast Drinking Water Directive includes a parametric value of 500 ng/L for total PFAS in drinking water [53].

Data reported by the non-governmental organization Pesticide Action Network Europe (PAN Europe) classify TFA as a “forever chemical” and document 63% of bottled water samples exceeded the limits proposed under the revised European Drinking Water Directive. Tap water concentrations averaged 740 ng/L, with a range spanning from below the detection limit (<20 ng/L) to 4100 ng/L. Bottled mineral and spring waters showed concentrations between non-detectable levels and 3200 ng/L, with a mean value of 278 ng/L [54].

## 4. Strengths and Limitations

This narrative review provides a focused and up-to-date synthesis of new evidence on trifluoroacetic acid (TFA) in wine, integrating environmental, analytical, exposure, and toxicological perspectives. A key strength of this work is the combined assessment of peer-reviewed studies and recent European monitoring reports, which allows for a comprehensive interpretation of both quantitative data and temporal trends. Furthermore, the review contextualizes wine within the broader environmental cycle of TFA, highlighting its role as an integrative matrix reflecting long-term contamination processes.

Despite these strengths, some limitations should be acknowledged. The number of peer-reviewed studies specifically addressing the presence of TFA in wine remains limited and geographically inconsistent, with incomplete coverage of major wine-producing regions. Furthermore, the intrinsic physicochemical properties of TFA pose significant analytical challenges, and the lack of harmonized methodologies reduces comparability between studies and may contribute to the variability of reported concentrations.

A further limitation concerns the reliance on selected grey literature sources, including regulatory documents, technical reports, and non-governmental monitoring reports. These sources provide timely information on an emerging contaminant for which peer-reviewed wine-specific data remain scarce; however, they may lack the methodological transparency, peer-review process, and detailed quality-control reporting required for full comparability with scientific studies. For this reason, grey literature data were interpreted cautiously and used primarily to contextualize and support findings from peer-reviewed evidence.

Another limitation is the heterogeneity of analytical methods used for TFA detection and quantification. Differences in sample preparation, detection limits, recovery rates, and matrix-effect correction precluded formal standardization or meta-analytic pooling; therefore, reported concentrations were interpreted as indicative ranges rather than directly comparable estimates.

Comparative data across dietary and environmental matrices are also limited. Although available evidence suggests that wine may contain higher concentrations of trifluoroacetic acids (TFA) than other plant-based beverages, systematic multi-matrix investigations are still lacking, limiting the ability to contextualize wine-specific findings within the context of total dietary exposure.

Exposure assessment is also affected by uncertainty. Current estimates are based on simplified consumption scenarios and do not fully account for interindividual variability or cumulative intake from multiple sources. Furthermore, toxicological knowledge remains incomplete. Although existing studies indicate low acute toxicity, robust data on chronic exposure at low doses in humans are still lacking, reflecting the relatively recent emergence of TFA as contaminants of interest.

Finally, as a narrative review, this work does not follow a formal PRISMA-based systematic review protocol and does not include a quantitative meta-analysis. Consequently, no formal risk-of-bias assessment, screening flowchart, or exhaustive list of excluded records was produced. However, methodological transparency was strengthened by clearly reporting the search strategy, eligibility criteria, source categories, and rationale for including peer-reviewed literature together with selected regulatory and monitoring documents.

## 5. Conclusions

This review identifies trifluoroacetic acid (TFA) as an ultra-persistent and highly mobile contaminant, whose environmental accumulation is increasingly reflected in terrestrial food systems. Wine appears to be a particularly informative matrix, with TFA consistently detected in modern vintages and largely absent in historical samples, reflecting the increasing use and environmental release of fluorinated chemicals.

However, comparison with other beverages and plant-derived food matrices indicates that wine should not be considered a unique source of TFA exposure, but rather a potentially relevant contributor within a broader pattern of dietary intake involving beer, tea, herbal infusions, fruit juices, nectars, drinking water, and other plant-derived products.

The combination of increasing environmental concentrations, chronic dietary intake, and the lack of food-specific regulatory limits highlights the need for precautionary monitoring.

Given the increasing ubiquity of TFA and its continued input into the environment, further research is needed to better characterize their toxicological profile and potential health implications.

Future efforts should focus on the development of harmonized analytical methodologies, comprehensive monitoring across wine-growing regions, and integrated multi-matrix exposure assessment frameworks. In particular, specific toxicological and epidemiological studies are needed to clarify the effects of chronic dietary exposure. Future studies should also assess whether winery-related factors, such as processing aids, filtration materials, winery water, cleaning agents, and food-contact materials, may influence TFA levels in finished wine. At present, direct evidence supporting a relevant contribution of these factors is lacking. Similarly, current evidence does not support the hypothesis that naturally occurring grape constituents generate TFA during winemaking. The absence of food-specific guidance values for trifluoroacetic acid (TFA), including in wine, represents an important area for further risk-assessment development rather than evidence of an established health risk. Future regulatory discussions should be supported by harmonized analytical data, broader monitoring across wine-producing regions, and improved toxicological and exposure evidence.

In this context, wine can serve not only as a sentinel matrix for environmental contamination, but also as a key element in promoting the understanding and regulation of ultra-persistent PFAS in the food chain.

## Figures and Tables

**Table 1:** Reported TFA concentrations in wine and selected plant-derived beverages.

Beverage Matrix	Reported TFA Levels	Key Findings	Reference
Wine	Up to 260 µg/L in archived Baden wines; up to 620 µg/L in recent international wines	Marked increase after 2010; absent or undetectable before 1970 in archived wines	[33]
Beer	Up to 51 µg/L; median 6.1 µg/L	TFA detected in 104 beer samples from 23 countries; malt identified as the likely main source	[23]
Tea/herbal infusions	Median 2.4 µg/L	TFA detected as an unintended aqueous extract from plant material	[23]
Fruit juices and nectars	Reported in comparative beverage analysis	Included as plant-derived beverages in comparison with wine; generally lower than recent wines	[33]

Note: Reported values should be interpreted as indicative rather than directly comparable estimates because available studies differ in sampling design, analytical methods, geographical origin, detection limits, and matrix-effect correction.

**Table 2:** Regulatory and environmental occurrence of trifluoroacetic acid (TFA) in drinking water and bottled water across Europe.

Regulatory/Spatial Context	Reported Concentration or Threshold	References
Germany—drinking water	Health-based guidance value: 60 µg/L; recommended precautionary target: <10 µg/L	[49]
Netherlands—drinking water	Provisional benchmark value: 2.2 µg/L	[50]
European Union—REACH regulation	DNEL * for the general population: 0.042 mg/kg body weight/day	[43,51,52]
European Union—revised Drinking Water Directive	Parametric value for total PFAS: 500 ng/L	[53]
Europe—bottled water analyzed by PAN Europe	63% of samples exceeded the proposed limits under the revised EU Drinking Water Directive	[54]
Europe—tap water	Mean concentration: 740 ng/L; range: <20–4100 ng/L	[54]
Europe—mineral and spring waters	Range: non-detectable-3200 ng/L; mean concentration: 278 ng/L	[54]

* Derived No-Effect Level.

## Data Availability

No new data were created or analyzed in this study. Data sharing is not applicable to this article.

## Abbreviations

The following abbreviations are used in this manuscript:

TFA	Trifluoroacetic Acid
PFAS	Polyfluoroalkyl Substances
PMT	Persistent, Mobile, Toxic
vPvMF	Persistent, very Mobile
SANRA	Scale for the Assessment of Narrative Review Articles
HFCs	Hydrofluorocarbons
HFOs	Hydrofluoroolefins
DNELs	Derived No-Effect Level
REACH	Registration, Evaluation, Authorisation and Restriction of Chemicals
PFOA	Perfluorooctanoic Acid
GC–MS	Gas Chromatography–Mass Spectrometry
LC–MS/MS	Liquid Chromatography coupled to tandem Mass Spectrometry
HILIC	Hydrophilic Interaction Liquid Chromatography
nsIC–MS	non-suppressed Ion Chromatography–MS/MS
